# Correlation of *In  Vivo* Versus *In Vitro* Benchmark Doses (BMDs) Derived From Micronucleus Test Data: A Proof of Concept Study

**DOI:** 10.1093/toxsci/kfv189

**Published:** 2015-10-05

**Authors:** Lya G. Soeteman-Hernández, Mick D. Fellows, George E. Johnson, Wout Slob

**Affiliations:** *National Institute for Public Health and the Environment (RIVM), Bilthoven, The Netherlands;; ^†^AstraZeneca, R&D Alderley Park, Macclesfield, Cheshire SK10 4TF, United Kingdom; and; ^‡^Institute of Life Science, Swansea University Medical School, Swansea University, SA2 8PP Wales, United Kingdom

**Keywords:** *in vitro* micronucleus, TK6 cells, benchmark dose approach, genotoxic potency

## Abstract

In this study, we explored the applicability of using *in vitro* micronucleus (MN) data from human lymphoblastoid TK6 cells to derive *in vivo* genotoxicity potency information. Nineteen chemicals covering a broad spectrum of genotoxic modes of action were tested in an *in vitro* MN test using TK6 cells using the same study protocol. Several of these chemicals were considered to need metabolic activation, and these were administered in the presence of S9. The Benchmark dose (BMD) approach was applied using the dose-response modeling program PROAST to estimate the genotoxic potency from the *in vitro* data. The resulting *in vitro* BMDs were compared with previously derived BMDs from *in vivo* MN and carcinogenicity studies. A proportional correlation was observed between the BMDs from the *in vitro* MN and the BMDs from the *in vivo* MN assays. Further, a clear correlation was found between the BMDs from *in vitro* MN and the associated BMDs for malignant tumors. Although these results are based on only 19 compounds, they show that genotoxicity potencies estimated from *in vitro* tests may result in useful information regarding *in vivo* genotoxic potency, as well as expected cancer potency. Extension of the number of compounds and further investigation of metabolic activation (S9) and of other toxicokinetic factors would be needed to validate our initial conclusions. However, this initial work suggests that this approach could be used for *in vitro* to *in vivo* extrapolations which would support the reduction of animals used in research (3Rs: replacement, reduction, and refinement).

Short-term genotoxicity tests are generally utilized in cancer risk assessment in a qualitative manner for hazard identification, but here we explored their applicability for quantitative analysis and prediction of cancer potency. *In vitro* genotoxicity assays are designed to detect a wide-range of different types of genetic damage, where certain outcomes require follow-up testing. For instance, *in vivo* genotoxicity tests may be performed because they take into account factors such as toxicokinetic and toxicodynamic processes, so that more relevant inferences on the potential risk of chemical exposure in humans can be made. The choice of follow-up *in vivo* tests depends on the type of genotoxic damage detected from *in vitro* tests (ie, gene mutations or chromosomal aberrations). Generally, an *in vivo* MN test is often performed if the compound was found to induce chromosomal aberrations *in vitro*. If there are indications that the compound induces gene mutations *in vitro*, then the transgenic rodent mutation assay is performed in potential target tissues (Eastmond *et al.*, 2009). Depending on the regulatory body, a positive result in an *in vivo* (or *in vitro*) genotoxicity study can result in the substance to be forbidden as in the case for food additives or can trigger a 2-year cancer bioassay to determine the carcinogenic potential of substances, and/or for the derivation of a point of departure (POD) for further risk assessment. A carcinogenicity study generally involves a 2-year exposure to a chemical using 50 animals (rodents) per dose per sex with a minimal of 3 doses (OECD, 2008). Shortcomings of the 2-year cancer bioassay include the large number of animals used (typically 400 per species), the long time it takes to get the results, and the high cost (∼1 to several million euros depending on route of exposure; (Jacobson-Kram *et al.*, 2004). Worldwide efforts are being made to reduce the number of animals used in research and, at the same time, satisfy regulatory requirements in keeping the human population safe.

In *Toxicity Testing in the 21st Century* (NRC, 2007) the use of novel data streams, such as *in vitro* mutagenicity data of DNA-reactive chemicals is emphasized, as well as the need for developing the methodology for using them as primary data in human hazard assessment. Several efforts are exploring the possibility of quantitatively using data from genetic toxicology studies for use in human health risk assessment (Gollapudi *et al.*, 2013; Hernandez *et al.*, 2011, 2012; Johnson *et al.*, 2014a,b; MacGregor *et al.*, 2014a,b; Soeteman-Hernandez *et al.*, 2015). These studies showed that *in vivo* genotoxicity studies provide more information than just the presence or absence of genotoxic potential for a given compound. The doses required to achieve a given genotoxic response in an *in vivo* MN test were found to differ considerably among substances. These equipotent doses, estimated as Benchmark doses (BMDs), were found to correlate with the doses resulting in a given level of carcinogenic response (Hernandez *et al*., 2012, 2012; MacGregor *et al*., 2014a,b; Soeteman-Hernandez *et al*., 2015). These studies suggest that the genotoxic potency assessed in an *in vivo* MN test might be used as a predictor of the carcinogenic potency of the same compound. This is conceivable given that the *in vivo* MN test measures the induction of chromosomal aberrations, a process that is generally considered to be strongly associated with carcinogenesis (Bonassi *et al.*, 2011). MN is commonly used as a biomarker of chromosomal damage, genome instability, and cancer risk in humans. There is preliminary evidence that MN frequency in peripheral blood lymphocytes is predictive of cancer risk (Bonassi *et al.*, 2011) and is used as an indicator of early genetic effects for instance as a result of occupational exposure to polycyclic aromatic hydrocarbons (Wang *et al.*, 2012), pesticides (Bolognesi *et al.*, 2011) or in cancer patients (Iarmarcovai *et al.*, 2008) as a biological marker for the efficacy of a chemo-preventive regime (Rosin, 1992).

In this study, we focus on the question to what extent *in vitro* genotoxicity tests could provide information on the *in vivo* genotoxic and carcinogenic potency of chemicals. A preliminary *in vitro* MN study with human lymphoblastoid (AHH-1) and Chinese Hamster fibroblast (V79) cell lines showed that after treatment with 17 -β-oestradiol (E_2_), bisphenol-A (BPA), and Rotenone, the BMDL_10_s for *in vitro* MN and the most sensitive tumor endpoint were in both cases ranked as E_2_>BPA>>Rotenone (Hernandez *et al.*, 2013). Even though these compounds are aneugens and the number of compounds was only 3, these results provided a first indication of the applicability of this methodology for the potential for deriving carcinogenic potency information from *in vitro* MN studies. To further explore this, we selected 20 compounds from those that were examined by Hernandez *et al.* (2012) and Soeteman-Hernandez *et al.* (2015), and for which a correlation was found between the *in vivo* MN BMDs and the cancer BMDs. These 20 chemicals were subjected to an *in vitro* MN tests using TK6 cell line, with the purpose of investigating whether they correlate with the earlier obtained BMDs from *in vivo* MN tests and from carcinogenicity studies (Hernandez *et al*., 2012). If BMDs from *in vitro* genotoxicity tests could provide information on the carcinogenic potency of compounds, this might be highly useful in improving test strategies and in supporting the reduction of animals used in research (3Rs: replacement, reduction, and refinement).

## MATERIALS AND METHODS

### 

#### Test Compounds

**FIG. 1.**
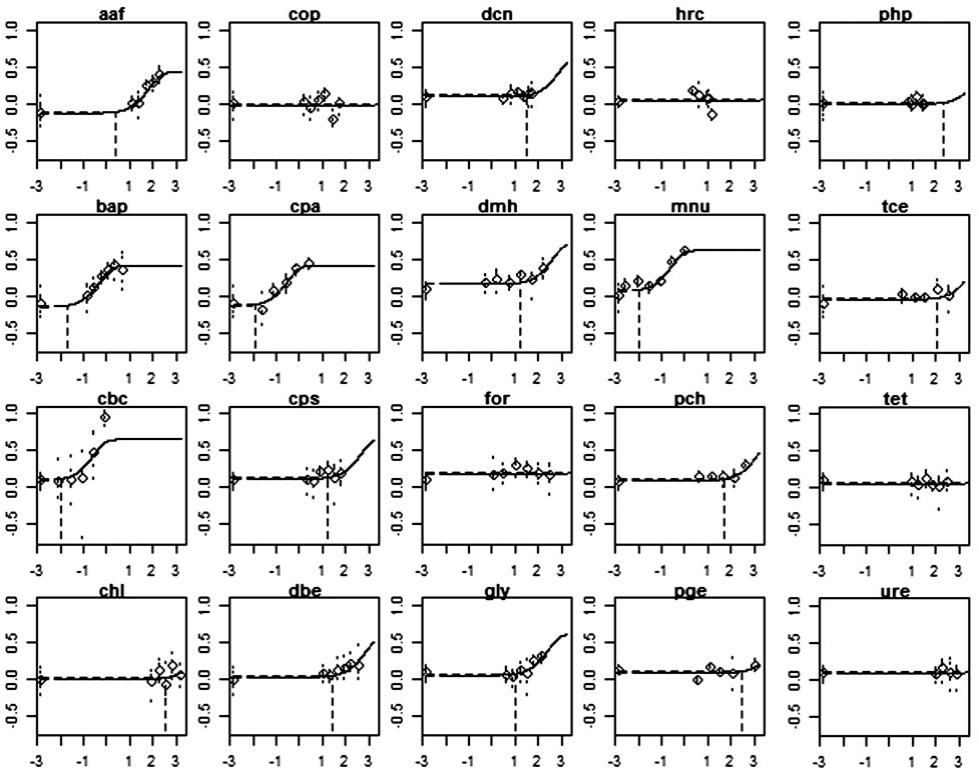
Best fitting curves for each compound, resulting from a fitted (4-parameter) exponential model to the combined dataset assuming that each compound had its own background response and potency, but that the shape parameters are the same among compounds. For each compound, a BMD was derived with respect to its control. The vertical dashed line indicates the BMD05 (μg/ml) for the fitted curve. x-axes represent log10 of dose (scaled to obtain dose = 1 as being the maximum dose value, for numerical reasons). Controls are plotted at the value −6. y-axes represent log10 of fraction (%) of micronuclei in 1000 cells scored. The circles represent the geometric mean of the percent *in vitro* micronucleus for each concentration tested. Abbreviation: BMD, Benchmark dose.

The list of 20 compounds that were tested in the *in vitro* MN test is presented in Table 1, together with the abbreviations used in this paperarticle. This table also shows the concentrations used for each compound, and whether or not S9 (metabolic activation) was applied. The concentrations to be tested in each compound, as well as the requirement for metabolic activation was based on previously published genotoxicity and cytotoxicity data and from range-finding experiments performed at AstraZeneca UK (Figure Fig. 1). Although the chemicals chosen were those already known to have yielded in vivo MN BMDs that correlated well with tumor BMDs, as demonstrated in Table 2, compounds selected also included those that were equivalent or negative for in vivo MN (cbc, dmh, pge, and tce) and for carcinogenicity (chl, hrc, and cps) using traditional methods. In addition, there were 7 compounds (cop, dbe, dcn, hrc, php, tet, and ure) that were negative in the in vitro MN and positive in the in vivo MN, and one 1 compound (tce) was positive in the in vitro MN and negative in vivo MN using the pairwise statistical significance methods for defining positives and negatives (Table 2).

**TABLE 1.** List of Compounds, Abbreviations, Study Number, and Concentrations Tested

Compound	Abbreviation	Cas. No.	Concentration (ug/ml)	S9
2-Acetylaminofluorene	aaf	53-96-3	0, 14, 28, 56, 112, 223	Yes
2-Acetylaminofluorene	aaf		0, 14, 28, 56, 112, 223	Yes
Benzo(a)Pyrene	bap	50-32-8	0, 0.16, 0.32, 0.63, 1.3, 2.5, 5	Yes
Benzo(a)Pyrene	bap		0, 0.16, 0.32, 0.63, 1.3, 2.5, 5	Yes
Chlorambucil	cbc	305-03-3	0, 0.00913, 0.0304, 0.0913, 0.304, 0.913	No
Chlorambucil	cbc		0, 0.00913, 0.0304, 0.0913, 0.304, 0.913	No
Chloral Hydrate	chl	302-17-0	0, 103, 207, 414, 827, 1654	No
Chloral Hydrate	chl		0, 103, 207, 414, 827, 1654	No
4-Chloro-o-phenylenediamine	cop	95-83-0	0, 1.8, 3.6, 7.1, 14, 29, 57	No
4-Chloro-o-phenylenediamine	cop		0, 1.8, 3.6, 7.1, 14, 29, 57	No
Cyclophosphamide	cpa	50-18-0	0, 0.028, 0.084, 0.28, 0.84	Yes
Cyclophosphamide	cpa		0, 0.028, 0.084, 0.28, 0.84, 2.8	Yes
P,P – dichlorodiphenyl sulfone	cps	80-07-9	0, 2.24, 4.48, 8.97, 18, 36, 72	No
P,P – dichlorodiphenyl sulfone	cps		0, 2.24, 4.48, 8.97, 18, 36, 72	No
1,2-Dibromoethane	dbe	106-93-4	0, 12, 23, 47, 94, 188, 376	No
1,2-Dibromoethane	dbe		0, 12, 23, 47, 94, 188, 376	No
Decalin	dcn	91-17-8	0, 3.5, 7, 14, 28, 41, 55	No
Decalin	dcn		0, 3.5, 7, 14, 28, 41, 55	No
1-2 Dimethylhydrazine	dmh	306-37-6	0, 0.06, 0.018, 0.6, 0.18, 60, 180	No
1-2 Dimethylhydrazine	dmh		0, 0.06, 0.018, 0.6, 0.18, 60, 180	No
Chloroform	for	67-66-3	0, 1.19, 3.57, 11.9, 35.7, 119, 357	No
Chloroform	for		0, 1.19, 3.57, 11.9, 35.7, 119, 357	No
Glycidol	gly	556-52-5	0, 4.625, 9.25, 19, 37, 74, 148	No
Glycidol	gly		0, 4.625, 9.25, 19, 37, 74, 148	No
4-Hexylresorcinol	hrc	136-77-6	0, 2.4, 4.9, 9.78, 13, 16	No
N-Nitro-N-Methylurea	mnu	684-93-5	0, 0.0031, 0.0103, 0.031, 0.103, 0.31	No
N-Nitro-N-Methylurea	mnu		0, 0.0031, 0.0103, 0.031, 0.103, 0.31, 1.03	No
4-Chloroaniline hydrochloride	pch	20265-96-7	0, 49.2, 164, 492	No
4-Chloroaniline hydrochloride	pch		0, 4.92, 16.4, 49.2, 164, 492	No
Propylene Glycol Mono-T-Butyl Ether	pge	57018-52-7	0, 3.96, 13.2, 39.6, 132, 1320	No
Phenolphthalein	php	77-09-8	0, 9.54, 31.8	No
Phenolphthalein	php		0, 9.54, 31.8	No
Phenolphthalein	php		0, 7.96, 16, 32	No
Trichloroethylene	tce	79-01-6	0, 3.94, 13.1, 39.4, 131, 394	Yes
Trichloroethylene	tce		0, 3.94, 13.1, 39.4, 131, 394	Yes
1,1,2,2-Tetrachloroethane	tet	630-20-6	0, 11, 21, 42, 84, 168, 336	No
1,1,2,2-Tetrachloroethane	tet		0, 11, 21, 42, 84, 168, 336	No
Urethane	ure	51-79-6	0, 111, 223, 445, 891	No
Urethane (ure)	ure		0, 111, 223, 445, 891	No

Each row represents a replicate.

**TABLE 2.** Outcome of Tests Using the Traditional Methods for Determining a Positive Response in the *In Vitro* and *In Vivo* MN and Carcinogenicity Studies

Compound	*In Vitro* MN	*In Vivo* MN	Cancer	Source Cancer	IARC	Mode of Action
aaf	+	+^1^	+	CPD	−	Mutagenic hepatocarcinogen^8^
bap	+	+^2,3^	+	CPD	1	Mutagenic^9^ and clastogenic^10^ carcinogen
cbc	+	e^4^	+	CPD	1	Mutagenic^11^ carcinogen
chl	+	+	e	TR-502	3	Aneugenic^12^ carcinogen (interferes with tubulin assembly and shortens microtubules)
cop	E	+	+	TR-063	2B	Mutagenic^13^ carcinogen
cpa	+	+^5,6^	+	CPD	1	Clastogenic^10^ carcinogen
cps	+	+	−	TR-501	−	Noncarcinogen^14^
dbe	E	+	+	TR-086	2 A	Mutagenic^15^ carcinogen
dcn	−	+	+	TR-513	−	Mutagenic^16^ carcinogen (rat kidney specific, alpha2u-globulin)
dmh	+	e^7^	+	CPD	2A	Mutagenic^17^ and clastogenic^18^ carcinogen
for	+	+	+	TR-000 (67-66-3)	2B	Nongenotoxic^19^ carcinogen (cytotoxicity and regenerative hyperplesia)
gly	+	+	+	TR-374	2A	Mutagenic and clastogenic^20^ carcinogen
hrc	−	+	e	TR-330	−	Noncarcinogen^21^
mnu	+	+	+	CPD	2A	Mutagenic and clastogenic^22^ carcinogen
pch	+	+	+	TR-351	−	Noncarcinogen^23^
pge	−	−	+	TR-515	3	Nongenotoxic^24^ carcinogen (alpha2u-globulin)
php	−	+	+	TR-465	2B	Clastogenic^25^ carcinogen
tce	+	−	+	TR-002	2A	Nongenotoxic^26^ carcinogen (peroxisome proliferator/tumor promoter^27^)
tet	−	+	+	TR-027	3	Weak mutagenic^28,29^ carcinogen
ure	−	+	+	TR-510	2A	Mutagenic^30^ carcinogen

IARC, International Agency for Research on Cancer; CPD, carcinogenic potency database (http://potency.berkeley.edu/); MN, hematopoietic MN test; AB, abbreviation; TR, National Toxicology Program technical report; +, positive; −, negative; e, equivocal. ^1^(Asano and Hagiwara, 1992); ^2^(Vrzoc and Petras, 1997); ^3^(Shimada *et al.*, 1992); ^4^(Morita *et al.*, 1997); ^5^(Gorelick *et al.*, 1999); ^6^(Hatanaka *et al.*, 1992); ^7^(Meli and Seeberg, 1990); ^8^(Heflich and Neft, 1994); ^9^(Benford *et al.*, 2010); ^10^(Sobol *et al.*, 2012); ^11^(Mohamed *et al.*, 2009); ^12^(Fellows *et al.*, 2011); ^13^(Staedtler *et al.*, 1999);^14^(NTP, 2001); ^15^(Liu *et al.*, 2007); ^16^(NTP, 2005); ^17^(Newell and Heddle, 2004); ^18^(Ashby and Mirkova, 1987); ^19^(Butterworth *et al*., 1998); ^20^(Ikeda *et al.*, 2012); ^21^(NTP, 1988); ^22^(Johnson *et al*., 2009); ^23^(NTP, 1989); ^24^(NTP, 2004); ^25^(NTP, 1996); ^26^(Wilmer *et al*., 2014); ^27^(Tabrez and Ahmad, 2009); ^28^(Colacci *et al.*, 1989); ^29^(McGregor *et al.*, 1988); ^30^(Hernandez and Forkert, 2007).

#### *In* V*itro* MN Test

##### Metabolic activation (S9)

For treatments in the presence of exogenous metabolism, S9 from the livers of Aroclor 1254 treated rats was purchased from Molecular Toxicology Inc. (Boone, North CarolinaNC, USA) and stored frozen at a temperature of (-65°C or below until use. On the day of use, S9 mix was prepared by the addition of culture medium containing cofactors for NADPH generation to the S9 fraction. A final S9 concentration 2% vol/vol was used.

##### Test agents

The test agents were dissolved in dimethyl sulphoxide (DMSO) before use. All chemicals and reagents were purchased from Sigma Aldrich. A list of compounds, abbreviations and the concentrations used are presented in Table 1. *N*-Nitroso-*N*-Methylurea and benzo[*a*]pyrene were used as positive controls.

##### Cell culture

The TK6 cell line (known in early publications as H2BT) is a subclone of WI-L2 established in 1968 (Levy, 1968). The cells used for this study were obtained as a gift from Swansea University in 2009. In house karyotypic analysis in 2010 showed these TK6 cells to have a modal chromosome number of 47 and a stable composite karyotype of 47 XY, +der13t(13;22) -14 +der14t (14;20) der 21 (21,3) .

TK6 cells were routinely cultured in Roswell Park Memorial Institute 1640 (RPMI) medium (Invitrogen, Paisley, UK) supplemented with 10% heat-inactivated donor horse serum, 2 mmol/L l l-glutamine, 2 mmol/L l sodium pyruvate, 200 IU/mlL penicillin, and 200 µg/mL ml streptomycin (R10). Cells were grown at 37°C in a humidified atmosphere of 5% CO_2_ in air and had an average doubling time of 15–16 hours. Cells were generally maintained at between approximately 2 ×x 104 and 1 ×x 106 cells//mlL (Molloy *et al.*, 2010).

##### Treatment

All the *in vitro* MN tests were performed in the same lab. Treatment exposure was for 3 h in the presence or absence of S9 as appropriate for each test agent. 1 × 10^6^ TK6 cells were suspended in 4 ml RPMI, containing 2.5% heat-inactivated donor horse serum. The test compound or solvent control solutions were added at 1% vol/vol. Quadruplicate solvent control and duplicate test compound cultures were prepared. Following treatment, the cells were centrifuged, washed once, and resuspended in R10 at a final cell concentration 1 × 10^5^ cells/ml. Cultures were incubated for approximately 40 h. Microscope slides were prepared by centrifuging at least 1 × 10^5^ cells in a Cytospin 3 (ShandonTM) centrifuge (800 rpm [100 × g] for 8 min) and fixed with methanol. Slides were stained with acridine orange. All identified MN were confirmed by eye to be separate and within the cytoplasm, to have intact cytoplasmic membrane and to be less than one-third of the diameter of the main nucleus. Where possible, a total of at least 1000 cells per culture were scored. The response for dose-response analysis was %cells with MN. In Table 2, *in vitro* MN positives are shown based on pairwise statistical significance testing with a *P* < .05. Table 3 summarizes the experimental design from the *in vitro* MN.

**TABLE 3.** Summary of Experimental Design Used in the *In Vivo* MN Studied in Figure 2 and Carcinogenicity Studies in Figure 3

MN (Genotoxicity Endpoint)							Carcinogenicity (Tumor Endpoint)						
compound	Mouse Strain	Sex	Route	Tissue	Duration Exposure (days)	Sampling time (hours)	Mouse Strain	Sex	Route	Exposure Time (wks)	Duration Experiment (wks)	Tissue	Tissue Lesion
Aaf	bdf1	m	ip	blood	1	48	bcn	m	feed	96	104	Bladder	Carcinoma
Bap	bdf1	m	gav	blood	1	48	b6c	f	feed	96	104	Forestomach	Squamous carcinoma
Cbc	bdf1	m	ip	bm	1	48	swiss	m	ip	26	78	Lymphoid system	Lymphoma
Chl	B6	m	ip	bm	3	24	b6c	m	gav	104	104	Liver	Hepatocellular carcinoma
Cop	cd1	m	ip	bm	2	48	b6c	m	feed	96	96	Liver	Adenocarcinoma
Cpa	cd1	m	ip	blood	1	48	swiss	f	ip	26	79	Lung	Malignant carcinoma
Cps	B6	m	ip	bm	3	24	b6c	f	feed	104	104	Skin	Sarcoma
Dbe	B6	m	inh	blood	175	24	b6c	f	gav	53	90	Stomach	Squamous carcinoma
Dcn	B6	m	inh	blood	91	24	b6c	f	inh	105	105	Uterus	Stromal polyp sarcoma
Dmh	cd1	m	gav	bm	1	1	swa	m	water	52	52	Hematopoetic system	Blood vessel angiosarcoma
For	B6	m	ip	bm	3	24	b6c	m	gav	93	93	Hematopoetic system	Lymphoma
Gly	P16	m	gav	blood	280	24	b6c	f	gav	104	104	Skin	Fibrosarcoma
Hrc	B6	m	ip	bm	3	24	b6c	m	gav	104	104	Adrenal gland	Pheochromocytoma
Mnu	balb	m	ip	blood	1	48	c3h	m	water	30	54	Stomach	Glandular adenocarcinoma
Pch	B6	m	gav	bm	3	24	b6c	m	gav	103	103	Liver	Hemangiosarcoma
Pge	B6	f	inh	blood	91	24	b6c	m	inh	104	104	Liver	Hepatoblastoma
Php	P16	f	feed	blood	42	24	b6c	f	feed	104	104	Hematopoetic system	Lymphoma
Tce	B6	m	gav	bm	3	24	b6c	m	gav	104	104	Liver	Carcinoma
Ure	B6	f	water	blood	91	24	b6c	f	water	104	104	Liver	Hemangiosarcoma

bm, bone marrow; gav, gavage; ip, intraperitoneal; inh, inhalation; m, male; f, female.

##### Cytotoxicity

For each treatment cytotoxicity was determined by calculation of a reduction in relative population doubling (RPD). Cell number was assessed on the day of sampling (1 day after treatment). Cultures giving RPD of less than 45% were considered to be excessively cytotoxic. This is in accordance with the suggested percent toxicity of 55% ± 5% in the recent study by Sobol *et al.* (1995) regarding the development and validation of an *in vitro* MN platform in TK6 cells (OECD, 2014). 

RPD was determined as:

$$\frac{\text{Number}\;\text{of}\;\text{Population}\;\text{doublings}\;\text{in}\;\text{treated}\;\text{cultures}}{\text{Number}\;\text{of}\;\text{Population}\;\text{doublings}\;\text{in}\;\text{control}\;\text{cultures}}\times \text{1}00$$

where

$$\begin{array}{c} \text{Population}\;\text{Doubling}=\\ \frac{\left[\text{log}\;(\text{cell}\;\text{number}\;\text{on}\;\text{day}\;\text{of}\;\text{sampling}/\text{initial}\;\text{cell}\;\text{number})\right]}{\text{log}\;\text{2}} \end{array}$$

The raw data are provided in the Supplementary Table 1.

##### BMD analysis

BMDs associated with the same benchmark response (BMR) (related to the same endpoint) are equipotent doses, and can thus be used to rank potencies of different compounds. In this article, the term potency is used in a relative sense only, and is not defined in an absolute sense (such as the “slope factor” in an LMS model fitted to cancer data).

BMD analysis was performed using PROAST, a dose-response modeling software package developed at the National Institute for Public Health and the Environment (RIVM) in the Netherlands (www.proast.nl). The genotoxicity data (% cells with MN out of 1000 cells scored) were analyzed as continuous data, and the 4-parameter exponential model was fitted to these data.

BMD analysis was performed on the *in vitro* MN data that were newly generated in this study. Similar to Hernandez *et al.* (2012) and Soeteman-Hernandez *et al.* (2015), the *in vitro* MN data were analyzed as one combined dataset, where compound was included as a covariate. The *in vivo* MN data, earlier analyzed in Soeteman-Hernandez *et al.* (2015), were reanalyzed taking compound as a covariate, as opposed to using individual dataset as a covariate. The compounds were found to differ significantly in background response (due to study differences) and potency (due to the compound differences), but otherwise the shape of the dose-response among different chemicals was found to be similar. The latter is a general phenomenon in toxicological dose-response data (Slob and Setzer, 2014), and was confirmed for the new *in vitro* MN data in this study (see Fig. 1). Fitting a single model to the combined dataset (with compound as covariate) results in smaller BMD confidence intervals as compared with fitting the model to each compound separately (Slob and Setzer, 2014). We did not take into account potential differences among replicate studies in the same compound (as available for part of the compounds, see Table 1). This means that the estimated dose-response for each compound (and hence the BMD) reflects an average of the replicate studies in the same compound.

The BMD results for the *in vivo* MN and the carcinogenicity dose-response data were obtained from Hernandez *et al.* (2012) and Soeteman-Hernandez *et al.* (2015). However, the *in vivo* MN data were re-analyzed with compound rather than individual dataset as a covariate. In this way, a single potency estimate (BMD confidence interval) was obtained for each compound, reflecting the average estimate over different dose-response datasets for that chemical, eg, relating to the 2 sexes, or the 2 tissues evaluated (blood, bone marrow).

It is important to note that, for the purpose of correlating *in vitro* MN potency to *in vivo* MN potency (or cancer potency), equipotent doses should not be estimated as single point estimates (BMDs) but rather as BMD confidence intervals, for various reasons. First, the BMD is only an estimate with a certain precision that may be good or poor, depending on the chemical’s dataset. Thus, a point that appears to be an outlier in the correlation plot could be so for biological reasons or simply because that point was an imprecise estimate. This distinction can be made visible by considering BMD confidence intervals rather than single BMD values. Another reason is that, in this way, compounds showing no or only a weak dose-response do not need to be omitted but can be taken into account in the analysis. These compounds will have a confidence interval with a finite lower bound and an infinite (or very large) upper bound. This interval tells us that the dose where the response is equal to the equivalent effect size (BMR) will be larger than the lower bound of the confidence bound (called BMDL). In other words, the specified increase in MN (=BMR) will not likely occur at a dose lower than the lower BMD confidence bound (=BMDL). Replacing such an infinite confidence interval with a single estimate of the BMD would be misleading, and not represent the information that is available for that dataset.

##### Choice of BMR

For deriving (equipotent) doses, it can be expected that the value of the BMR is not essential (as long as it is the same for all chemicals in the group). The reason is that the statistical analysis assumed the dose-responses to be parallel (against log-dose). This theoretical notion is confirmed by Bemis *et al.* (2015) who calculated BMD confidence intervals for various values of the BMR, resulting is similar correlations. We used a BMR of 5% change in mean response as compared with the controls, as this value was also used for the BMDs from the *in vivo* MN tests in our earlier study (Soeteman-Hernandez *et al*., 2015) and it is also a recommended BMR for continuous response data by the European Food Safety Authority (EFSA, 2009a,b). Just like this earlier study we used a BMR of 10% extra risk for carcinogenicity studies used because it is the most commonly used value of the BMR in dose-response characterization of quantal endpoints (EFSA, 2009a,b). When the BMDs in both systems are proportionally correlated (as will appear to be the case in our results) changing the BMR would only affect the proportionality constant (shift the correlation line), without changing the correlation (scatter around the line) as such.

It may be noted that the BMR for continuous data can also be defined in terms of the SD, the standard deviation of the within group variation. A BMR as a percentage change appears to better reflect the biological change in MN needed for an increased cancer risk than the BMR in terms of the within group SD. The latter definition of a BMR is subject to coincidental experimental heterogeneity or errors, including measurement errors (in the case of MN depending on the number of cells counted) A BMD for a given percent change is less sensitive to experimental conditions and errors, and appears a better measure for comparing equipotent doses/concentrations across endpoints (Johnson *et al*., 2014b).

##### Dose-response analysis

Dose response analysis was performed similarly as previously published (Soeteman-Hernandez *et al*., 2015). Briefly, for “continuous” dose-response data from the *in vitro* and *in vivo* MN test, data were analyzed by fitting the exponential model, which is one of the recommended models for continuous data (EFSA, 2009a,b) and known to be generally applicable to toxicity data:

$$y=a\left[c-\left(c-1\right)\exp\left(-bx^{d}\right)\right]$$

where *y* is the response (proportion of cells with MN) and *x* the dose. In fitting the model to the combined cluster of datasets, separate values for *a* (reflecting the background response at dose 0) and *b* (reflecting the potency of the chemical) are estimated for each compound in the dataset, whereas parameters *c* and *d* are kept constant over all datasets within the cluster analyzed. The within group variance was estimated separately for each compound as well. Please refer to Slob (2002) or Slob and Setzer (2014) for a more detailed discussion of this method.

For the “quantal” dose-response data from the carcinogenicity studies the log-logistic model was fitted.

$$y=\frac{a+(1-a)}{1+\exp[-c\log(x/b)]}$$

where *y* is the response (fraction of affected animals) and *x* the dose. Again, parameters *a* (reflecting the background response at dose zero), and *b* (reflecting the potency of the chemical) are estimated for each individual dose-response dataset, whereas (shape) parameter *c* is kept constant over all datasets within the analyzed cluster (See Soeteman-Hernandez *et al.* [2015] for more details).

##### Examining correlations between systems

**FIG. 2.**
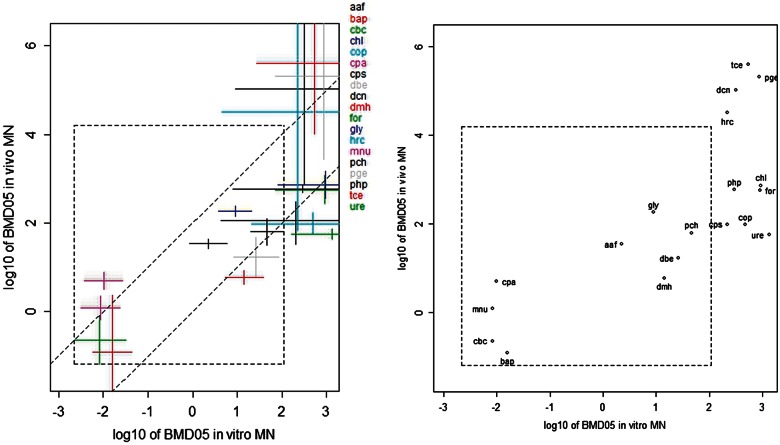
BMD confidence intervals for *in vivo* MN studies against those for *in vitro* MN studies. In both types of studies the BMD relates to BMR = 5%. The x-axis represents log10 of concentration in µg/ml, the y-axis log10 of dose in mg/kg/day. The 2 parallel lines have a slope 1, and were drawn by eye such that they span 2 orders of magnitude in vertical direction. See Table 1 for abbreviations of the associated individual compounds. Abbreviations: BMD, Benchmark dose; MN, micronucleus.

**FIG. 3.**
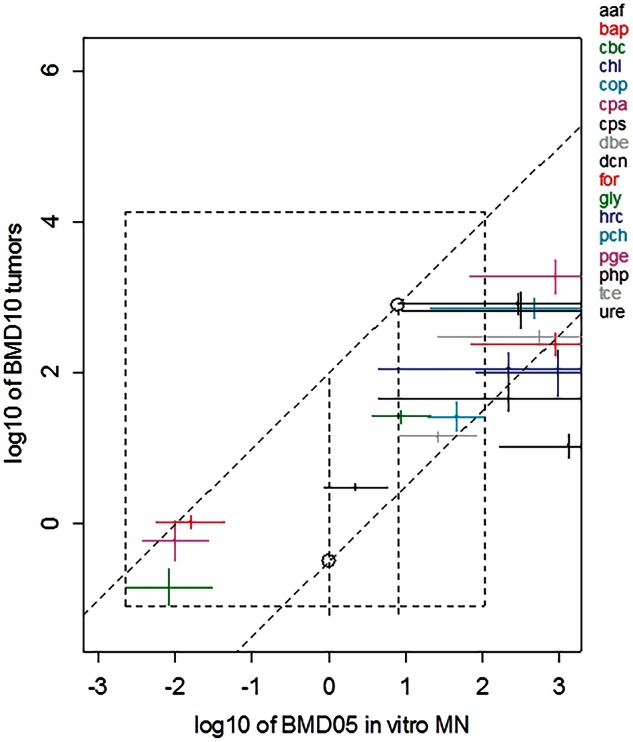
BMD10 confidence intervals from carcinogenicity studies (lowest found for malignant tumors in a single tissue) against BMD05s from *in vitro* MN test. The latter are the same as those in Figure 2. The x-axis represents log 10 of concentration in µg/ml, the y-axis log10 of dose in mg/kg/day. The 2 parallel lines roughly encompass the observed BMD confidence intervals as found in the test chemicals. By taking the lowest intersection point with the left vertical line and the highest intersection point with the right vertical line we obtain the uncertainty range for the predicted BMD10 for carcinogenicity (see the 2 plotted circles). In this way, both the prediction uncertainty related to the scatter in the correlation and the uncertainty in the BMD on the x-axis is taken into account. See Table 1 for abbreviations of the associated individual compounds. Abbreviation: BMD, Benchmark dose.

The usual way of quantifying a correlation is by deriving a correlation coefficient. However in our case, a substantial part of the chemicals resulted in one-sided infinite confidence intervals. Those chemicals definitely need to be included in examining the correlation, as they represent (in most cases) the weakly potent chemicals. These chemicals may provide important information: a weak potency in 1 system being associated with a high potency in the other would mean that this chemical does not comply with the overall correlation, which may have biological significance. Instead, correlation plots (*in vitro* MN vs *in vivo* MN in Fig. 2 and *in vitro* MN vs carcinogenicity in Fig. 3) were created by plotting their CIs (in both the x- and y-direction) related to matching chemicals, including the ones that resulted in (1-sided) infinite CIs. Inside the correlation plots we plotted a dashed box, indicating the largest finite BMDU (and lowest BMDL) of all intervals assessed, so that it is directly visible which CIs have infinite bounds (ie, when they cross outside the dashed box). As already noted, a deviating value (BMD) might be the result of low precision in a test system, and to make that possibility visible, the correlation between BMDs needs to be examined based on the complete BMD confidence intervals, taking both the lower (BMDL) and upper bound (BMDU) into account.

Instead of calculating a correlation coefficient we evaluated the observed correlations in another way. The BMDs in the 2 systems were plotted against each other on double-log scale. A linear relationship (with intercept zero) on the original scale translates into a straight line with unity slope in a double log-plot. Therefore, we drew 2 lines with unity slope (in the double log-plots) such that they encompass most of the BMD confidence intervals. If the individual chemicals are more or less randomly located between these 2 lines, this indicates that the relationship between the BMDs on y- and x-axis is (approximately) linear, in the sense that their values differ by a proportionality constant. Further, the vertical distance between both lines reflects an uncertainty margin related to the prediction of the BMD on the y-axis based on a given BMD on the x-axis. For instance, our previous analysis (Soeteman-Hernandez *et al*., 2015) showed that the cancer BMD can be predicted within around 2 orders of magnitude from a given value of the *in vivo* MN BMD. This uncertainty margin is in fact a measure of correlation: the better the correlation, the smaller the uncertainty margin. This visual way of establishing the correlation will suffice for the time being, as we are mainly interested in the question if information from *in vitro* or *in vivo* genotoxicity tests can at all be useful for predicting carcinogenic potency, and if so, for which type of chemicals (modes of action). A formal method for estimating the precision in that prediction may be developed at a later stage.

## RESULTS

### 

All of the *in vitro* MN TK6 dose-response data were analyzed as a single combined dataset using compound as a covariate, assuming that compounds differed in background response (due to different studies) and in potency, but not in the shape of the dose-response. The resulting curves associated with the fitted model to the combined dataset are individually shown for each compound in Figure 1. Visual inspection of the plots shows that the assumption of equal shapes was not violated, except possibly for the compound chlorambucil (cbc), where the observed response at the highest dose is not well described by the fitted curve. However, this dose group could also be an “outlier,” ie, the particular dose group might have differed from the other treatment groups in that study by some unknown experimental factor other than the dose (see Slob and Setzer, 2014, for a discussion of dose group outliers).

For most compounds 2 replicate studies were available. It was found that in some cases the dose-responses were somewhat dissimilar. In the analysis shown in Figure 1 this was ignored, and the BMD confidence intervals for each compound should thus be regarded as relating to the compound’s potency averaged over the 2 replicated studies.

From the 20 compounds with *in vitro* MN dose-response data, only 1 (the compound “tet”) resulted in a 2-sided unbounded confidence interval (meaning that the data are not informative enough to even decide whether the compound is very potent or not potent at all). Such an interval does not provide any information on the equipotent dose, and it was deleted in further analyses. From the remaining 19 compounds, 9 showed (2-sided) finite confidence intervals and 10 showed confidence intervals with an infinite upper bound (see Table 4). It is important to note that almost all the derived *in vitro* MN BMDL05 values (Table 4) were at concentrations where cytotoxicity was >90%.

**TABLE 4.** BMD05 Confidence Intervals for *In Vitro* and *In Vivo* MN Tests and BMD10 Confidence Intervals for Cancer per Compound

Compound	*In Vitro* MN Test (µg/ml)		*In Vivo* MN Test (mg/kg/day)		Cancer (mg/kg/day)	
	BMDL	BMDU	BMDL	BMDU	BMDL	BMDU
aaf	0.85	5.9	26.9	44.0	2.7	3.4
bap	0.006	0.04	0.001	2.36	0.9	1.3
cbc	0.0022	0.031	0.06	0.8	0.08	0.25
chl	82.4	Inf	360	1507	49.7	198
cop	21.1	Inf	55.8	166	522	960
cpa	0.0037	0.027	3.20	7.601	0.3	1.1
cps	4.39	Inf	32.3	296	30.4	66.0
dbe	8.36	83.9	5.96	47.2	12.3	16.7
dcn	9.03	Inf	723	Inf	381	1148
dmh	5.31	38.4	4.125	8.53	NA	NA
for	72.7	Inf	270	1184	169	347
gly	3.73	21.2	141	237	21.5	32.5
hrc	4.47	Inf	67.6	Inf	71.1	187
mnu	0.0031	0.023	0.633	2.27	NA	N/A
pch	20.2	108	32.1	122	16.3	41.3
pge	70.5	Inf	2780	Inf	1103	3167
php	7.92	Inf	478	715	567	1187
tce	26.8	Inf	10249	Inf	230	378
ure	163	Inf	43.2	74.6	7.5	15.2

#### In Vivo Versus In Vitro MN

Table 3 summarizes the experimental design from the *in vivo* MN studies. Table 4 shows the BMD confidence intervals for the *in vitro* MN studies against those for the *in vivo* MN studies on the same chemicals. Figure 2 shows the same results graphically. Note that decreasing values for the BMD indicates increasing potency. A proportional relationship between the BMDs in both systems translates into a line with unity slope in a double-log plot. The 2 parallel dashed lines in Figure 2 are unity slope lines drawn by eye. This indicates that, overall, the *in vivo* BMD approximately relates proportionally to the *in vitro* BMD. The distance between the 2 dashed lines in the vertical direction is 2 log10 units. Hence, the uncertainty in predicting the *in vivo* BMD from the *in vitro* BMD approximately would be somewhat more than 2 orders of magnitude (as some of the chemicals are just outside the 2 dashed lines). None of the 19 chemicals was found in the more extreme top-left or bottom-right areas of the plot, ie, no chemicals were found with low potency the *in vitro* and high potency in the in vivo MN test, or *vive versa*.

#### Carcinogenicity Versus In Vitro MN

Table 3 summarizes the experimental design from the carcinogenicity studies. Figure 3 shows the correlation between the *in vitro* BMD confidence intervals and the cancer BMD confidence intervals earlier obtained from carcinogenicity studies (Soeteman-Hernandez *et al.*, 2015). For 2 of the chemicals tested in the *in vitro* MN test (dmh, mnu) no adequate tumor BMDs could be derived (due to short exposure durations in the available carcinogenicity studies in both dmh and mnu, and lowest dose with high response in dmh). When incidence data related to various types of lesions were available in the same compound, we first selected the datasets related to malignant tumors observed in a single tissue (if available), and from those we selected the one resulting in the lowest BMD. The rationale for focusing on a single type of cancer lesions is that BMDs associated with different types of lesions (reflecting different stages of the carcinogenicity process) may not represent equipotent doses, which is essential for the research question here as with our previous study (Soeteman-Hernandez *et al*., 2015). Given that i*n vivo* MN potencies were found to correlate with cancer potencies (Soeteman-Hernandez *et al*., 2015), the correlation found in Figure 3 was not unexpected. For the 9 compounds with finite BMD confidence intervals resulting from the *in vitro* MN test, the correlation with the tumor BMDs is good. For the 10 chemicals resulting in *in vitro* BMDs with infinite upper bounds the associated tumor BMDs had finite BMD CIs. Because the latter were in the higher end of the range (ie, low potency) their exact relative position could not be predicted based on the *in vitro* potency. All that they could predict was that the cancer potency would be relatively low. The 2 parallel sloped lines roughly encompass the observed BMD confidence intervals as found in the test chemicals. By taking the lowest intersection point with the left vertical line and the highest intersection point with the right vertical line we obtain the uncertainty range for the predicted BMD10 for carcinogenicity (see the 2 plotted circles in Fig. 3). In this way, both the prediction uncertainty related to the scatter in the correlation and the uncertainty in the BMD on the x-axis is taken into account.

## DISCUSSION

Genetic toxicity studies have been generally used in a qualitative yes/no fashion to assess whether a compound is genotoxic or not. However, recent efforts have explored ways of quantifying the compound’s genotoxic potency by examining suitable metrics based on dose-response analysis of genotoxicity data. The BMD approach was shown to be a suitable method for examining *in vitro* and *in vivo* genotoxicity studies for methyl methanesulfonate (MMS), ethyl methanesulfonate (EMS), 1-methyl-1-nitrosourea (MNU) and 1-ethyl-1-nitrosourea (ENU) (Gollapudi *et al*., 2013; Johnson *et al., 2*014b). In this study, we applied the BMD approach for estimating equipotent doses in *in vitro* MN tests. By correlating these *in vitro* BMDs to cancer BMDs we found a first indication that not only *in vivo* MN tests provide useful information on the carcinogenic potency of compounds (Johnson *et al*., 2014b), but that this may also hold for *in vitro* MN tests in TK6 cells. It is evident that data for more compounds are needed, including those that are mutagenic rather than clastogenic, that are generally negative for *in vivo* or *in vitro* MN yet are carcinogens, that require metabolic activation, and non-carcinogens. Nevertheless, we have shown that potency information can be obtained from the *in vitro* MN test in TK6 cells, and further research is warranted to further validate our findings.

In general, the BMD confidence intervals resulting from the *in vivo* studies are smaller than those from the *in vitro* studies (Fig. 3). In particular, there were 6 chemicals that resulted in an infinite BMD upper bound in the *in vitro* studies, but in a finite BMD upper bound in the *in vivo* studies (Table 4 and Fig. 2). These results indicate that the (current) *in vivo* MN test is more sensitive in the statistical sense: the probability of detecting compounds with relatively weak genotoxicity potency is greater in the *in vivo* MN study than in the *in vitro* MN studies in TK6 cells. This could be due to differences in study design. For example, the number of replicates in the *in vivo* MN tests used for *in vivo* BMDs was usually larger than that in the *in vitro* tests as performed in the present study.

Normally, it may be expected that much of the observed scatter in Figure 2 is due to the fact that the *in vitro* test does not account for the toxicokinetic processes (absorption, distribution, metabolism, and elimination) that may be relevant for evoking the genotoxic response *in vivo.* Note that toxicokinetic processes could have an impact in both directions. For example, metabolic activation may result in a relatively high potency *in vivo*, whereas limited absorption after oral exposure could lead to relatively low *in vivo* potency, as compared with *in vitro*. Piersma *et al.* (2008) used toxicokinetic information to evaluate the observed scatter when correlating *in vivo* to *ex vivo* reproductive BMDs, illustrating that toxicokinetic information could be used in a further analysis of the correlation between *in vivo* and *in vitro* BMDs. However, for many of the 19 compounds comprising the correlation plot in Figure 2 very little (quantitative) toxicokinetic information is available, and an analysis as performed by Piersma *et al.* (2008) was not feasible. However, it is likely that toxicokinetic information could explain part of the scatter in Figure 2.

Given the correlation that was found between the BMDs from the *in vitro* and the *in vivo* MN test, and given the earlier reported correlation between *in vivo* MN and cancer BMDs (Hernandez *et al*., 2012), it could be expected that the BMDs from the *in vitro* MN would also correlate to the cancer BMDs. As shown in Figure 3, such a correlation was indeed present for the 17 compounds for which adequate data were available (for 2 of the 19 chemicals no suitable carcinogenicity data were available). The compound urethane (ure) seems to deviate most from the overall correlation scatter. Urethane is negative in mammalian cells (mouse lymphoma assay and *in vitro* MN studies) and it remains uncertain as to whether urethane an Ames positive compound is given that this has never been confirmed (Kirkland *et al.*, 2014). Although metabolism via CYP2E1 is required, no evidence for mutagenic activity was reported with urethane when S9 from rats induced with CYP2E1 was used in the Ames test (Burke *et al.*, 1994). It is not clear why urethane is “missed” by mammalian cell tests (Kirkland *et al*., 2014).

There are several ways of performing *in vitro* to *in vivo* extrapolations. One approach focuses on biokinetic modeling with the purpose of relating to make appropriate adjustments for binding and other factors affecting the free concentration of the compound and converting the associated *in vitro* concentration into human equivalent *in vivo* concentrations (Blaauboer, 2010; Yoon *et al.*, 2014). This approach is applicable when sufficient toxicokinetic information with regards to the substance in question is available. For cases where such chemical-specific toxicokinetic information is not available, one may adopt another approach and try to find empirical relationships between *in vitro* concentrations and *in vivo* doses, as we did in this study. For instance, Walum *et al.* (2005) established a relationship between *in vitro* cytotoxic concentrations and *in vivo* acute lethal doses using 50 reference substances, and showed a high predictability of *in vitro* cytotoxic concentrations for human acute toxic doses. A similar approach was taken by the Registry of Cytotoxicity database assembled by the Federal Institute for Risk Assessment (BfR) which contains *in vitro* IC50 values and rodent LD50 values for a total of 347 substances. Spielmann *et al.* (1999) analyzed these data and showed that the IC50 values could be used for predicting the LD50 value. The Interagency Coordinating Committee on the Validation of Alternative Methods (ICCVAM) recommended the correlation model from Spielmann *et al*. (1999) as a tool for predicting an LD50 value to be used as a starting dose for the Acute Toxic Class method (TG 423) or the Up-and-Down Procedure (TG 425). Computer simulations showed that using *in vitro* cytotoxicity assays to estimate an LD50 as a starting dose could potentially reduce animal use by 28% for acute oral toxicity testing, and by 50% for nonclassified substances (ICCVAM, 2006; OECD, 2010). We foresee that *in vitro* genotoxicity test may have a similar impact and applicability.

### 

#### Predicting Potencies Based on Correlations Between Systems

To illustrate how a BMD (and its uncertainty range) in 1 system can be predicted from the BMD in the other system, and how this might be used in risk assessment, consider a hypothetical chemical X found in a food product. An *in vitro* MN test is available for this chemical, indicating that it is genotoxic. A risk manager is interested in the cancer risk for consumers of the contaminated food product. However, neither a carcinogenicity study nor an *in vivo* genotoxicity study is available for that chemical. The lower and upper intersection points of the vertical lines with the sloped lines (indicated by the circles in Fig. 3) may be considered as the lower and upper bound of the uncertainty range for the predicted cancer BMD10. Here, the 2 sloped lines have intercepts—0.5 and 2 on the log-scale, so the lower and upper bound are 10^−^^0.5 ^= 0.32, and 10^2.4 ^= 250 mg/kg, respectively. Thus, the BMD10 for carcinogenicity is predicted to be somewhere in the range between 0.32 and 250 mg/kg/day. This result could be used as a reference for deriving a Margin of Exposure between the lower/upper bound of the predicted BMD for carcinogenicity and the estimated exposure in the human subpopulation. If the margin of exposure (MOE) with the lower bound of the predicted BMD is much larger than 10 000 it might be concluded that there is no reason of concern (Barlow *et al.*, 2006). If the MOE with the upper bound of the predicted BMD would be smaller than 10 000 this would indicate a reason of concern. In intermediate cases, a conclusive answer might not be possible. This illustration demonstrates one possible application of this methodology in instances where risk management need to make cancer-risk related decisions in the absence of carcinogenicity data.

#### Limitations

This proof of concept approach informs only with regards to the POD but does not inform on the subsequent steps needed for the derivation of an acceptable risk of chemicals for humans. There are currently many discussions on how different the low-dose extrapolation approaches with the same POD can lead to very different lower acceptable exposure levels. Acceptable exposure levels can be orders of magnitude apart depending on whether the linear low-dose cancer slope factor approach was used or the threshold reference dose/uncertainty approach. Mode of action information is therefore crucial for identifying compounds with modes of action not considered to be directly DNA reactive such as cytotoxicity (for in Table 2) where a nonlinear threshold approach is applicable, instead of a linear approach (Butterworth *et al.*, 1998). Mode of action can also inform with regards to human relevance as seen with rodent-specific peroxisome proliferators (tce in Table 2) (Wilmer *et al.*, 2014), and alpha2u-globulin-rat kidney specific tumors (dcn and pge in Table 2) (NTP, 2004, 2005). There are also examples where mode of action could be the same, such as MNU and ENU, both being DNA reactive alkylating agents, however the potency, DNA adduct spectrum and mutation spectrum are different, so more detailed mechanism of action information may be required (Doak *et al.*, 2007; Johnson *et al.*, 2009, 2014b). The *in vitro* MN can distinguish between aneugens and clastogens if methods such as fluorescence *in situ* hybridization are included in follow-up work (Hernandez *et al*., 2013). The *in vitro* MN alone is insufficient for a short-term strategy. Nevertheless, we foresee our approach being part of an integrated *in vitro* high throughput screening strategy, as outlined in *Toxicity Testing in the 21st Century* (NRC, 2007), where mode action and human relevance information can be supplemented by other *in vitro* screening methods and reducing animal testing.

Overall, we provide a proof-of-principle of the applicability of using *in vitro* MN data for predicting the *in vivo* genotoxic potency of a compound, as well as for predicting the cancer potency of a compound. This finding warrants further research with larger sets of compounds, with various MOAs. We are currently collaborating with international government agencies (Health Canada, US FDA) and international organizations (ILSI/HESI GTTC) to expand our database with more chemicals and to refine the methodology. The server www.MutAIT.org has been established as a data repository for collecting *in vitro* and *in vivo* genotoxicity and carcinogenicity data.

## Supplementary Material

Supplementary Data
