# Supplementary material for: Correlation of In Vivo Versus In Vitro Benchmark Doses (BMDs) Derived From Micronucleus Test Data: A Proof of Concept Study
Source: Toxicol Sci. 2015 Oct 5;148(2):355–67. doi: 10.1093/toxsci/kfv189 (PMC4659532; doi:10.1093/toxsci/kfv189)
Supplement: Supplementary Data [file supp_148_2_355__index.html]

Correlation of in vivo versus in vitro benchmark doses (BMDs) derived from micronucleus test data: A proof of concept study — Correlation of In  Vivo Versus In Vitro Benchmark Doses (BMDs) Derived From Micronucleus Test Data: A Proof of Concept Study — Correlation of In  Vivo Versus In Vitro Benchmark Doses (BMDs) Derived From Micronucleus Test Data: A Proof of Concept Study — Supplementary Data 

# Correlation of *In  Vivo* Versus *In Vitro* Benchmark Doses (BMDs) Derived From Micronucleus Test Data: A Proof of Concept Study

## Supplementary Data

files

- Supplementary Data - xlsx file
